# Commentary: Increased Prefrontal Activity with Aging Reflects Nonspecific Neural Responses Rather than Compensation

**DOI:** 10.3389/fnint.2020.00009

**Published:** 2020-02-21

**Authors:** Madeline A. Gregory

**Affiliations:** Department of Psychology, University at Buffalo, The State University of New York, Buffalo, NY, United States

**Keywords:** aging, fMRI, memory, compensation, maintenance, prefrontal activity

Over the past several decades research on memory in aging has made numerous important discoveries regarding age-related differences in brain activation during cognitive tasks. Chief among them is the observation first made by Grady et al. (1994) that healthy older adults show increased prefrontal cortex (PFC) activation, accompanied by decreased occipital cortex activation, during visual processing. This pattern of age-related increase in frontal activity coupled with decrease in posterior activity has been observed in many different cognitive domains, including facets of memory such as working memory (Rypma and D'Esposito, 2000; Grossman et al., 2002), and episodic memory (Anderson et al., 2000; Cabeza et al., 2004).

An fMRI study by Davis et al. (2008) defined this phenomenon as the posterior–anterior shift in aging (PASA). They suggested PASA was a compensatory mechanism and were able to show that performance was positively correlated with age-related increases in PFC activity and negatively correlated with age-related decreases in occipital activity, during both an episodic retrieval task and a visual perception task. However, an alternative explanation for increased PFC reliance is that increased activity might instead reflect reduced efficiency or specificity of neural responses, ultimately signaling a decline in PFC functionality (Park et al., 2004; Raz and Rodrigue, 2006; Nyberg et al., 2012).

Morcom and Henson (2018) examine task-related PFC activation via fMRI using a novel approach: the multivariate Bayes (MVB). MVB estimates the patterns of activity that best predict memory performance using the spread of multivariate responses, thus allowing for a direct comparison of theories (Morcom and Johnson, 2015). Tasks consisted of a long-term memory (LTM) encoding task and a short-term memory (STM) maintenance task. MRI data from both PFC regions and Posterior Visual Cortex (PVC) regions were examined.

The LTM task involved an in-scanner associative encoding portion in which subjects were presented with a background scene followed by a superimposed object. Participants were then asked to create a story linking the object to the scene. The associative retrieval portion took place outside the scanner, during which participants were asked to verbally recall the scene paired with a given object. The STM task involved visual stimuli consisting of either red dots, yellow dots or blue dots. Set size was varied so that any of 1, 2, or 3 of the dot displays moved in a single direction that had to be remembered. Memory load was varied so that participants had to recall a dot display from one of 1, 2, or 3 stimuli ago.

Performance of both LTM and STM tasks produced age-related increases in PFC activation, consistent with the findings of earlier studies. However, with increasing age, PFC activity was *less* predictive of memory performance. MVB analyses showed that, in both PFC and PVC, the spread of multivariate responses predicting memory outcomes was particularly reduced later in life; in comparing a joint PVC-PFC model to a PVC-only model, it was found that PFC did not “boost” the prediction of memory performance. The authors suggest that this age-related increase in PFC activity reflects either less efficient or less specific neural function, both of which are consistent with the idea of brain maintenance: increased PFC activity reflects the efforts of the older brain to maintain, albeit unsuccessfully, youth-like function.

These findings help explain the previously defined links between cognitive reserve (CR) and compensation. Several studies have shown that higher levels of CR lead to increased compensatory capacity. Steffener et al. (2011) showed that this increased compensation was associated with diminished task performance. Consistent with brain maintenance accounts, this suggests that “the use of an alternate [neural] network may maintain as opposed to improve performance” (Barulli and Stern, 2013). Brain maintenance is conceptualized by “individual differences in the manifestation of age-related brain changes,” which allows some to show “little or no age-related cognitive decline” (Nyberg et al., 2012). The findings by Morcom and Henson (2018) might also explain the discrepancy in which age-related frontal overrecruitment is found with cross-sectional analyses, while frontal underrecruitment is found with longitudinal analyses (Nyberg et al., 2010). Overrecruitment of the PFC may reflect brain maintenance processes which delay the onset of cognitive decline, but which may not be sustainable as aging progresses.

The generalizability of these results to other cognitive tasks is unclear. Consider the nature of the memories the participants were asked to recall: in the LTM task, participants were asked to verbally recall the background scene that had been paired with a given test object “in terms of detail or gist” (Morcom and Henson, 2018). In the STM task, participants adjusted a pointer until it matched the direction of motion of the target dot display, a continuous judgement. Neither task had a unique correct response. There is therefore considerable potential variability in the precision of subjects' responses, which could represent differential contributions of familiarity vs. recollection processes (Yonelinas et al., 1999). Given that recollection relies on the PFC and the hippocampus while familiarity relies on parahippocampal regions, it is difficult to conclude that all participants were equally engaged in PFC recruitment during the retrieval process, for which MRI data was either not examined or unavailable (Yonelinas, 2002).

Furthermore, the authors do acknowledge that their results point to some support for PASA. In the STM task only, the relative involvement of PFC vs. PVC voxels did increase with age, suggesting that PASA may be task-specific rather than task-general (Morcom and Henson, 2018). However, this is not necessarily evidence that greater PFC involvement is compensatory.

It is also possible that, while the increased PFC activity may be non-specific or inefficient, it could still be useful in encoding memories or using abilities which rely more heavily on the PFC, such as working memory or inhibition. For example, it has been shown that on the go/no-go task, older adults exhibit similar behavioral performance on no-go trials compared to younger adults, suggesting older adults maintain response inhibition abilities, which are are heavily PFC-dependent (Hsieh et al., 2015).

Given the very different implications of each theory of age-related changes in PFC activity, it is imperative to definitively determine their functional significance. MVB appears to be a promising approach through which this can be investigated. Standard statistical approaches reveal only that PFC activity increases, leaving ample room for speculation. And even if, as this paper suggests, it is brain maintenance rather than compensation driving this increased PFC activity, this may still represent an adaptive cortical response intended to delay age-related cognitive decline.

## Author Contributions

The author confirms being the sole contributor of this work and has approved it for publication.

### Conflict of Interest

The author declares that the research was conducted in the absence of any commercial or financial relationships that could be construed as a potential conflict of interest.
